# Early hemodynamic assessment using NICOM in patients at risk of developing Sepsis immediately after emergency department triage

**DOI:** 10.1186/s13049-021-00833-1

**Published:** 2021-01-28

**Authors:** Steve B. Chukwulebe, David F. Gaieski, Abhishek Bhardwaj, Lakeisha Mulugeta-Gordon, Frances S. Shofer, Anthony J. Dean

**Affiliations:** 1 Department of Emergency Medicine, Advocate Sherman Hospital, Elgin, IL USA; 2 Department of Emergency Medicine, Sidney Kimmel Medical College at Thomas Jefferson University, 1025 Walnut Street; 300 College Building, 19107 Philadelphia, PA USA; 3 Department of Internal Medicine, Division of Pulmonary and Critical Care Medicine, Cleveland Clinic, Cleveland, OH USA; 4 Department of Obstetrics and Gynecology, Hospital of the University of Pennsylvania, Philadelphia, PA USA; 5 Department of Emergency Medicine, Perelman School of Medicine, University of Pennsylvania, Philadelphia, PA USA

**Keywords:** Severe sepsis, Noninvasive monitoring, Cardiac output, Cardiac index, Heart rate, Lactate

## Abstract

**Background:**

One factor leading to the high mortality rate seen in sepsis is the subtle, dynamic nature of the disease, which can lead to delayed detection and under-resuscitation. This study investigated whether serial hemodynamic parameters obtained from a non-invasive cardiac output monitor (NICOM) predicts disease severity in patients at risk for sepsis.

**Methods:**

Prospective clinical trial of the NICOM device in a convenience sample of adult ED patients at risk for sepsis who did not have obvious organ dysfunction at the time of triage. Hemodynamic data were collected immediately following triage and 2 hours after initial measurement and compared in two outcome groupings: (1) admitted vs. dehydrated, febrile, hypovolemicdischarged patients; (2) infectious vs. non-infectious sources. Receiver operator characteristic (ROC) curves were calculated to determine whether the NICOM values predict hospital admission better than a serum lactate.

**Results:**

50 patients were enrolled, 32 (64 %) were admitted to the hospital. Mean age was 49.5 (± 16.5) years and 62 % were female. There were no significant associations between changes in hemodynamic variables and patient disposition from the ED or diagnosis of infection. Lactate was significantly higher in admitted patients and those with infection (p = 0.01, p = 0.01 respectively). The area under the ROC [95 % Confidence Intervals] for lactate was 0.83 [0.64–0.92] compared to 0.59 [0.41–0.73] for cardiac output (CO), 0.68 [0.49–0.80] for cardiac index (CI), and 0.63 [0.36–0.80] for heart rate (HR) for predicting hospital admission.

**Conclusions:**

CO and CI, obtained at two separate time points, do not help with early disease severity differentiation of patients at risk for severe sepsis. Although mean HR was higher in those patients who were admitted, a serum lactate still served as a better predictor of patient admission from the ED.

## Background

Sepsis, defined as a dysregulated inflammatory response syndrome to an infectious trigger, causing acute organ dysfunction, is one of the leading causes of death worldwide [1–3]. The mortality is greater than 15 % for sepsis and greater than 20 % for septic shock [2]. One factor leading to the high mortality rate seen in sepsis is the subtle, dynamic nature of the disease, which can lead to delayed detection and under-resuscitation. Because of the natural progression of sepsis, patients often present to the Emergency Department (ED) dehydrated, febrile, hypovolemic, hyperdynamic, and vasoconstricted. Initial pro-inflammatory effects act to increase capillary permeability causing fluid shifts into the interstitial space, resulting in further hypovolemia, and, in a subset of patients, depressed myocardial function. Varying hemodynamic profiles can be present during the proximal phase of sepsis in the ED [4]. Later, anti-inflammatory stages of the syndrome can cause immunomodulatory dysfunction and multiorgan failure [3]. Since the treatment of hypovolemia and myocardial depression involves different and at times competing modalities, an understanding of a patient’s specific hemodynamic profile early in the treatment course may improve outcomes.

Numerous studies have examined various biomarkers that track the body’s response to bacterial infection, systemic inflammation, tissue perfusion, and systemic oxygen delivery, and have identified them as significant predictors of severity of illness [5–7]. The most promising of these biomarkers is lactate and elevated lactate levels are associated with myocardial dysfunction, hypoperfusion, and mortality [8, 9]. However, a patient with a significantly elevated lactate level can have supranormal, normal, or depressed hemodynamic function [8, 9]. Thus, it has been suggested that other non-invasive means of prognosticating patients at risk for severe sepsis-associated hemodynamic deterioration are needed. To date, few studies have examined non-invasive means of tracking the hemodynamic changes in sepsis.

While pulmonary artery catheterization (PAC) historically has been the most studied method of monitoring hemodynamics, there are risks associated with PAC placement and limitations to the information obtained. A recent literature review regarding the use of PAC in sepsis patients by Karanikolas et al. found that there is no significant benefit in regards to outcomes [10]. Other techniques such as a transthoracic echocardiography, thoracic bioimpedance, exhaled CO_2_ measured by Fick’s principle, and arterial pulse contour have been assessed with variable agreement to PAC measurement [3, 11–13]. The Noninvasive Cardiac Output Monitor (NICOM; Cheetah Medical) uses bioreactance to determine cardiac output (CO) [14]. Bioreactance (dΦ) is measured by a change in the phase of a voltage signal sent from an electrode on one side of the chest to another electrode on the contralateral side. Peak rate of change is proportional to peak aortic flow. Thus, stroke volume (SV) can be calculated by the following formula, SV = C x VET x (dΦ/dt)_max_. C is a constant defined by the manufacturer and VET is the ventricular ejection time determined from the NICOM device by analysis of the electrocardiographic signals. Once SV is determined, that value can be used to compute CO. Cardiac Index (CI) can also be calculated if the patient’s height and weight are obtained. Although the NICOM has been studied in perioperative settings and intensive care units (ICU), relatively few studies have looked at its use in the non-traumatic ED patient [15–21]. Furthermore, there are no studies that use the NICOM as a way to prognosticate which patients presenting to the ED without obvious signs of acute organ injury are at greatest risk for developing severe sepsis.

This is a pilot study of NICOM to determine whether the hemodynamic parameters it provides can be used in triage or in a serial fashion to predict severity of disease in stable-appearing patients at risk for sepsis on ED presentation. Initial triage NICOM values obtained as soon as possible after ED triage as well as their change at a second time point two hours later were used to test the following three hypotheses: (1) NICOM values recorded over the first two hours in the ED, while stable patients often remain in the waiting room prior to initiation of treatment, will predict admission to the hospital when comparing admitted vs. discharged patients; (2) NICOM values recorded over the first two hours in the ED will differ in patients with an infectious vs. non-infection source; and (3) given the association of lactate with organ dysfunction and mortality in sepsis, NICOM values recorded over the first two hours in the ED will correlate with serum lactate levels in their efficacy to predict patient disposition from the ED.

## Methods

### Study Design and setting

This study was a prospective, observational, convenience sample study of the NICOM during a 4-month enrollment period from October 27th, 2014 to February 23rd, 2015.

### Population

The study was performed in the ED at an urban quaternary care center in a large metropolitan area.

Patients presenting to the ED and triaged as an Emergency Severity Index (ESI) 2 or 3, suggesting they can wait between 20 minutes and 2 hours to begin treatment, were eligible for enrollment if they met the following inclusion criteria:

1. Age ≥ 18 years;
2. At least 2 of the 3 SIRS criteria measured at triage (Temperature < 96.8°F or > 100.4°F; Heart rate (HR) > 90 beats per minute (BPM); Respiratory rate (RR) > 20 breaths/minute) were present;
3. An automatic lactate order was generated by the ED Advanced Triage Protocol (ATP), which required an ESI classification of ESI 2 or 3, the presence of at least 2 SIRS criteria in triage, AND a chief complaint consistent with infection. The presence of at least two triage SIRS criteria and one of the 50 pre-identified chief complaints that suggest they may have a focus of infection automatically prompts an order for a serum lactate through the HUP ED ATP [Appendix]. The goal of the ATP process is to expedite a serum lactate result to help risk stratify patients with possible sepsis in a patient population that includes patients who have the potential to decompensate during the period when they are waiting for treatment to begin..

Patients were excluded from the study if any of the following were present:

1. Assigned ESI 1, requiring immediate treatment by the clinical team;
2. Assigned either ESI 4 or 5, categorizing them as “stable to wait” for several hours for treatment to begin and assigned to be seen in the Fast Track section of the ED;
3. Altered mental status;
4. Inability to obtain informed consent;
5. Non-English speaking.

Patients who met inclusion criterion 3, the ED ATP, generated a text message to the research team and their records were reviewed. Patients who met all inclusion and exclusion criteria during a time when study personnel were available were approached for enrollment in the study and informed consent was obtained. Patient enrollment, chart review, and data collection was performed by a single observer (SBC). The study protocol was approved by the University of Pennsylvania’s Institutional Review Board prior to beginning patient enrollment.

### Demographics, historical, Triage, and Laboratory Data

Patient demographics including age, sex, race, weight, and height were collected. Triage data collected included temperature, HR, blood pressure, and RR. Comorbidities collected included a history of diabetes, coronary artery disease, congestive heart failure, chronic obstructive pulmonary disease, cancer, human immunodeficiency virus status, organ transplantion, systemic lupus erythematosus, smoking, and surgery in the month prior to triage presentation. Laboratory values collected include serum lactate, complete blood count, and chemistry panels.

### NICOM Data Collection

After informed consent was obtained, coincident with obtaining of a blood specimen to assess triage serum lactate level, enrolled patients were placed in a semi-recumbent position with the head of the bed at 45° and their feet fully extended. The NICOM sensors and blood pressure cuff were applied to the patient according to the manufacturer’s specifications. The device was allowed to calibrate for 3–5 minutes, at which point single values for the following data were collected and automatically stored: time, CO, CI, HR, blood pressure, mean arterial pressure (MAP), total peripheral resistance, total peripheral resistance index, SV, and stroke volume index (SVI). The pads were removed after the first measurements were obtained. After two hours, the same pads were replaced on the patient in approximately the same locations, and the process was repeated to obtain a second set of NICOM values. The values collected from the NICOM device were not available to the treating clinicians and were not used as part of patient management.

### Clinical outcomes

In the primary outcome, NICOM values obtained at time zero and two hours were compared in patients who were admitted to the hospital vs. those who were discharged from the ED. NICOM values were also compared in patients who had an infectious source vs. those who did not have an infectious source. Patients with a diagnosis of infection were defined by examining the charts of all patients enrolled in the study for ED physician documentation that suggested suspected infection, an order for blood cultures, use of antibiotics in the ED, and need for hospital admission. Patient classification was conducted without knowledge of the NICOM values obtained at time zero and two hours later.

### Statistical analysis

An analysis of variance in repeated measures (RM-ANOVA) was used to compare differences over time in the NICOM variables of CO, CI, SV, and HR in each of the two outcome groupings (admitted vs. discharged; infectious source vs. non-infectious source). The NICOM HR was included in the analysis to determine if the post-triage measurement of the NICOM specific values of CO, CI, and SV provide risk stratification information when compared to the triage HR. Additionally, receiver operator characteristic (ROC) curves were created to compare the area under the curve (AUC) and determine whether the initial NICOM CO, CI, SV, and HR values or the change in these measurements at the second time point predict hospital admission better than a serum lactate. SAS (Version 9.4, SAS Institute, Cary, NC), NCSS (Version 8.0, NCSS LLC, Kaysville, UT), and SPSS (Version 17.0, International Business Machines Corp., Armonk, NY) software were used to perform the statistical analysis. *p* < 0.25 was considered to be significant for interactions in the RM-ANOVA. A 2-sided p-value of *p* < 0.05 was considered to be significant in other analyses.

## Results

Approximately 100 text messages for patients meeting the ATP criteria were sent to study personnel during times when one was available to enroll potential patients and the potential patients’ ED records were reviewed. Fifty patients were consented and enrolled during the study period; the mean age was 49.5 (± 16.5) years; 62 % were female; and 56 % were African American. Cancer (14/50; 28 %) was the most common comorbidity, followed by Diabetes Mellitus (10/50; 20 %). Of the 50 patients, 32 (64 %) were admitted to the hospital from the ED; 14 (28 %) were diagnosed as SIRS without evidence of infection, 24 (48 %) as infection or sepsis, 11 (22 %) as severe sepsis and 1 (2 %) patient as having septic shock. A higher percentage of septic vs. non-septic patients were admitted to the hospital. Patients admitted from the ED were significantly older (55.2 vs. 39.5 years old, *p* = 0.001); more likely to be male (50 % vs. 17 %, *p* = 0.02); and more likely to have a history of cancer (38 % vs. 11 %, *P* = 0.05) when compared to those who were discharged (Table 1). Patients with an infectious source were significantly older that those without an infectious source (52.0 vs. 42.8 years old, P = 0.02). ATP lactate orders were generated on 100 % (50/50) patients; 74 % (37/50) had a lactate value drawn immediately after the order was generated; the mean lactate for this group was 1.5 ± 0.8 mmol/L.

**Table 1:** Patient characteristics

	All Patients		Admitted		Discharged		*P* Value	Infectious Source		Non-Infectious Source		*P* Value
Number (% of enrolled)	*N* =50		*N* =32 (64)		*N* =18 (36)			*N* =30 (60)		*N* =20 (40)		
Age, y	49.5	± 16.2	55.2	± 13.1	39.5	± 16.7	**0.001**	54.0	± 14.0	42.8	± 17.3	**0.015**
Female sex, (%)	31	(62)	16	(50)	15	(83)	**0.020**	17	(57)	14	(70)	0.341
African American, (%)	28	(56)	17	(53)	11	(61)	0.426	18	(60)	10	(50)	0.459
Caucasian, (%)	20	(40)	14	(44)	6	(33)	0.426	11	(37)	9	(45)	0.459
Asian, (%)	1	(2)	0	(0)	1	(6)	0.426	0	(0)	1	(5)	0.459
Weight (kg)	84.8	± 26.3	89.4	± 27.7	76.8	± 22.1	0.105	87.5	± 25.7	80.9	± 27.4	0.393
Height (in)	65.7	± 4.4	66.3	± 4.7	64.7	± 3.6	0.195	66.3	± 4.7	65.0	± 3.7	0.300
**Patient History**												
CAD, (%)	1	(2)	1	(3)	0	(0)	0.449	0	(0)	1	(5)	0.216
Cancer, (%)	14	(28)	12	(38)	2	(11)	**0.046**	11	(37)	3	(15)	0.095
CHF, (%)	2	(4)	1	(3)	1	(6)	0.674	1	(3)	1	(5)	0.768
COPD, (%)	8	(16)	7	(22)	1	(6)	0.131	6	(20)	2	(10)	0.345
Diabetes mellitus, (%)	10	(20)	7	(22)	3	(17)	0.659	6	(20)	4	(20)	1.000
HIV, (%)	3	(6)	3	(9)	0	(0)	0.180	3	(10)	0	(0)	0.145
Lupus, (%)	1	(2)	1	(3)	0	(0)	0.449	1	(3)	0	(0)	0.409
Smoking history, (%)	3	(6)	2	(6)	1	(6)	0.921	2	(7)	1	(5)	0.808
Surgery in past month, (%)	3	(6)	3	(9)	0	(0)	0.180	3	(10)	0	(0)	0.145
Transplant, (%)	4	(8)	3	(9)	1	(6)	0.633	2	(7)	2	(10)	0.670

Data are presented as mean ± standard deviation, or count (percentage). *CAD* indicates, coronary artery disease; *CHF* congestive heart failure; *COPD* chronic obstructive pulmonary disease; *HIV* human immunodeficiency virus

Admitted patients had a higher triage RR (22 vs. 19 breaths per minute, P = 0.01) and serum lactate (1.7 ± 0.8 vs. 0.9 ± 0.3 mmol/L, *P* = 0.01) when compared to discharged patients (Table 2). Patients with an infectious source had a significantly higher lactate than those without an infectious source (1.7 ± 0.9 vs. 1.0 ± 0.4 mmol/L, *P* = 0.01).

**Table 2:** Triage, Laboratory Data and Outcomes

	All Patients		Admitted		Discharged		*P* Value	Infectious Source		Non-Infectious Source		*P* Value
Number (% of enrolled)	*N* =50		*N* =32 (64)		*N* = 18 (36)			*N* = 30 (60)		*N* =20 (40)		
**Initial Triage Vitals**												
Temperature, F	100.4	± 1.9	100.1	± 1.9	100.9	± 1.9	0.147	100.3	± 1.9	100.5	± 2.0	0.646
Heart rate, per min	118.5	± 15.4	120.9	± 15.3	114.2	± 15.0	0.140	120.5	± 16.0	115.4	± 14.2	0.254
SBP, mmHg	135.0	± 25.1	136.9	± 27.9	131.6	± 19.3	0.472	132.4	± 27.6	138.9	± 20.8	0.375
DBP, mmHg	78.3	± 11.5	79.2	± 13.0	76.9	± 8.2	0.509	76.7	± 10.8	80.8	± 12.3	0.220
Respiratory Rate, per min	21.2	± 4.4	22.3	± 4.6	19.2	± 3.1	**0.012**	21.6	± 4.5	20.6	± 4.2	0.397
**Laboratory Values**												
WBC, x 10^9^/L	11.6	± 8.1	12.2	± 9.5	10.3	± 3.6	0.462	12.5	± 9.7	10.2	± 4.3	0.344
Hgb, g/dl	12.6	± 1.8	12.4	± 2.0	13.0	± 1.5	0.322	12.2	± 2.1	13.3	± 1.1	0.065
Creatinine, mg/dL	1.26	± 1.1	1.26	± 0.9	1.26	± 1.5	0.981	1.28	± 1.0	1.24	± 1.4	0.913
Lactate, mmol/L (37 pts)	1.48	± 0.8	1.68	± 0.8	0.93	± 0.3	**0.010**	1.69	± 0.9	1.02	± 0.4	**0.014**
**Other Characteristics**												
ED length of stay, hours	8.63	± 7.85	10.32	± 9.13	5.62	± 3.31	**0.041**	10.16	± 9.23	6.33	± 4.44	0.091
Hospital length of stay, days	N/A	N/A	5.71	± 5.94	N/A	N/A	N/A	5.42	± 5.10	7.28	± 10.05	0.530
ICU, (%)	3	(6)	3	(9)	N/A	N/A	N/A	2	(7)	1	(5)	0.808
Mortality, (%)	2	(4)	2	(6)	0	(0)	0.279	2	(7)	0	(0)	0.239

Data are presented as mean ± standard deviation, or count (percentage). SBP indicates systolic blood pressure; DBP, diastolic blood pressure; WBC, white blood cell count;

*ICU* intensive care unit. ICU stay was recorded within current admission. Mortality was recorded up to 30 days after enrollment

**Fig. 1:**
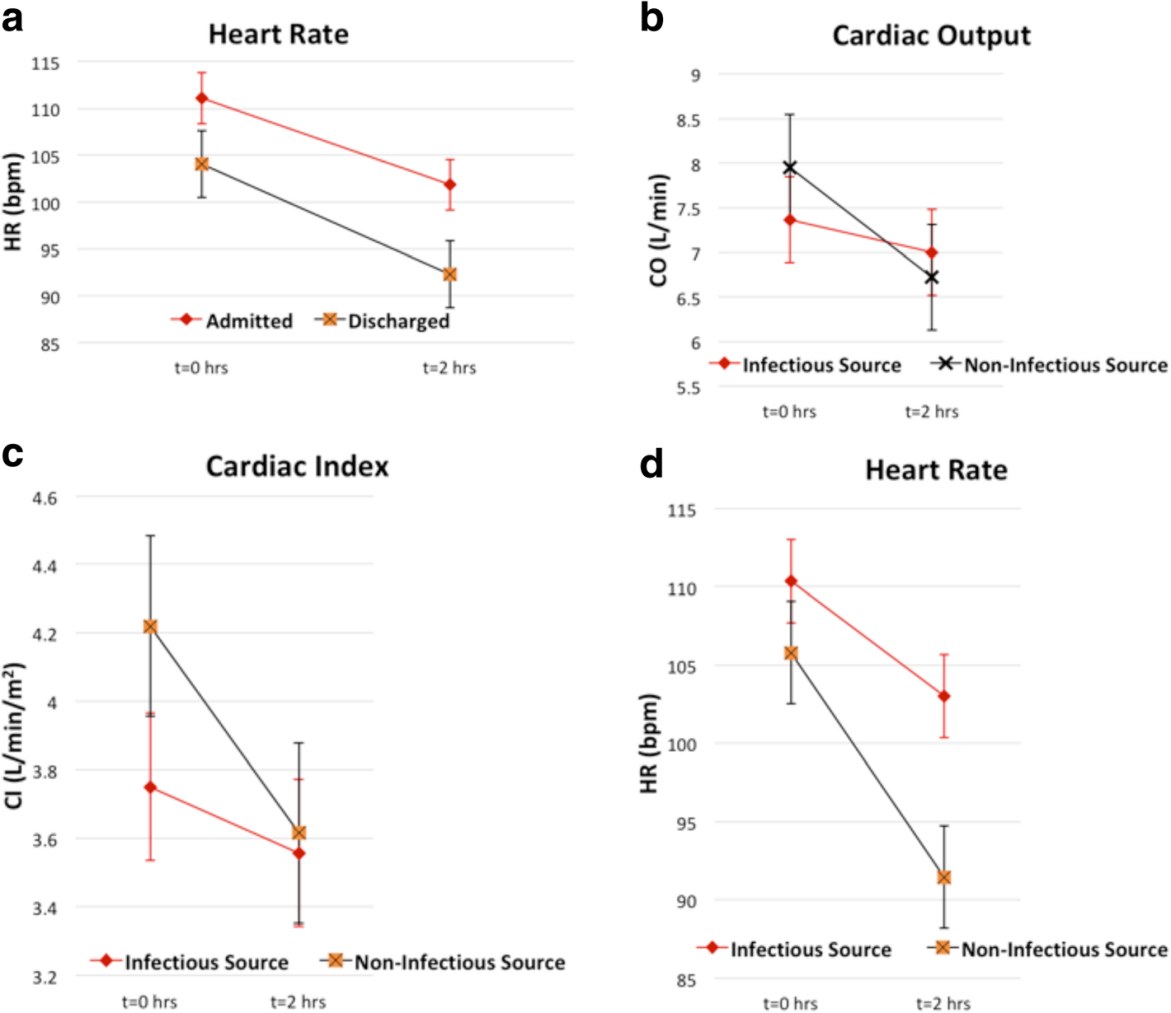
Repeated measures analysis of variance. **a**. HR values at time zero and two hours in patients who were admitted and discharged from the ED. There was no significance in the association between the change in HR between t=0 and t=2 hours with patient disposition (F = 0.62, *P* = 0.435). There was a significant difference in the mean HR when patients were grouped by disposition (*p* = 0.043). **b**. CO values at time zero and two hours in patients who had an infectious source and those without an infectious source. There was a significant association between the change in CO between t=0 and t=2 hours with infection (F = 2.48, *P* = 0.122). There was no significant difference in the mean CO when patients were grouped by source (*p* = 0.804). **c**. CI values at time zero and two hours in patients who had an infectious source and those without an infectious source. There was a significant association between the change in CI between t=0 and t=2 hours with infection (F = 2.82, *P* = 0.100). There was no significant difference in the mean CI when patients were grouped by source (*p* = 0.293). **d**. HR values at time zero and two hours in patients who had an infectious source and those without an infectious source. There was a significant association between the change in HR between t=0 and t=2 hours with infection (F = 5.32, *P* = 0.026). There was a significant difference in the mean HR when patients were grouped by source (*p* = 0.044). All data represented as means with error bars equal to 95% confidence interval. When associating NICOM values at time zero and two hours with another dependent variable (ie. patient disposition, LOS, or infectious source) a P-value of less than 0.25 was taken to be significant. Otherwise a *p*-value of less than 0.05 was used. CO indicates, cardiac output; CI, cardiac index; HR, heart rate

The average time from ED triage to 1st NICOM values was 38.0 ± 17.4 minutes and the range was 17–120 minutes. No patients received fluid resuscitation between the time zero and time 2-hour NICOM measurements. The mean values for CO, CI and HR at time zero were 7.60 L/min, 3.93 L/min/m^2^ and 109 BPM and at time two hours were 6.88 L/min, 3.58 L/min/m^2^ and 98 BPM respectively. In the RM-ANOVA, there were no significant associations between the change in CO, CI, or HR between time zero and time two hours with patient disposition from the ED (admitted vs. discharged; data not shown). However, there was a significant difference in the mean HR of patients (averaged between time zero and time two hours) who were admitted vs. discharged, 106 vs. 98 BPM, respectively (*p* = 0.04) (Fig. 1a). In patients with an infectious source, the change in CO and CI between time zero and time two hours was not statistically significant while the change in HR was (*p* = 0.12, 0.10, and 0.03 respectively) (Fig. 1b-d). SV increased significantly from time zero to time two hours, which is reflected in the fact that HR changes during this interval were significant while CO and CI changes were not (results not shown). Further, a significant difference was observed in the mean HR of patients with vs. without an infectious source (107 vs. 99 BPM; *P* = 0.04).

**Fig. 2:**
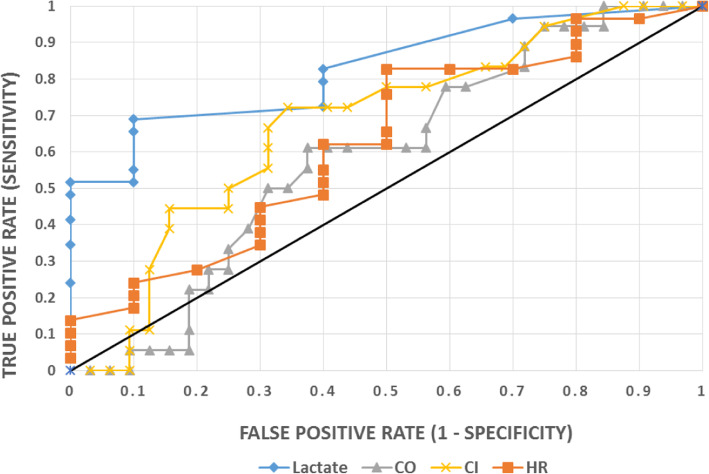
Receiver operator characteristic curves of lactate, CO, CI, and HR at time zero. Empirical ROC curve. Area under the curve values as follow: Lactate 0.828; CO 0.588; CI 0.677; HR 0.626. CO indicates, cardiac output; CI, cardiac index; HR, heart rate

An ROC analysis using initial lactate, comparing the 37 patients who had lactate measured immediately to the total cohort of 50 patients, and time zero values for CO, CI, and HR was used to determine the likelihood of a patient being admitted from the ED (Figure 2). The AUC (± 95 % Confidence Intervals) for lactate was 0.83 [0.64–0.92] compared to 0.59 [0.41–0.73] for CO, 0.68 [0.49–0.80] for CI, and 0.63 [0.36–0.80] for HR. The ROC analysis using the difference in CO, CI, and HR values yielded no significant findings. When the analysis was limited to the 37 patients who had lactate, CO, CI, and HR measured initially, the AUCs for CO and CI decreased to 0.35 ± 0.10 and 0.29 ± 0.10 respectively.

## Discussion

In this study examining a cohort of stable patients at risk for the development of sepsis after Emergency Department triage, we found that there were no significant associations between the change in CO, CI, or HR between time zero (immediately after triage) and time two hours and patient disposition from the ED (admitted or discharged to home). Similarly, there were no significant associations between the change in CO or CI, between time zero (immediately after triage) and time two hours and the presence or absence of an infectious source; the change in HR from time zero to time two hours, on the other hand, was significantly different between those with an infectious and those with a non-infectious source. Further, patients admitted to the hospital vs. those discharged and those with vs. those without infectious diagnoses had higher mean heart rates during the first two hours of their ED stays. Finally, we found that the initial lactate level ordered from triage correlated much better with the likelihood of admission when compared to changes in CO, CI, SV, or HR. Since both CO and CI are directly related to HR, this suggests that in this cohort of patients the NICOM CO and CI measurements may not be better than taking a simple triage HR followed by serial HRs during the first hours of care. In addition, in this cohort of patients, changes in SV to preserve CO and CI did not add appreciable information to that obtained from changes observed in HR alone. Additionally, the mean HR was shown to be the value that differentiated admitted patients from discharged patients and patients with an infectious source from those with a noninfectious source. This further suggests that, in the initial ED setting, for stable patients triaged as ESI 2 and 3, the NICOM device may not be better than a simple HR measurement to prognosticate disease severity. This study also provided evidence that lactate was a better predictor of patient disposition compared to CO, CI, and HR. Thus, given the poor predictive value of the NICOM values at triage and hour two post-triage, serum lactate obtained soon after triage remains one of the few reliable early prognosticators in stable patients at risk for development of sepsis.

Patients with a high suspicion for sepsis and obvious acute organ dysfunction at the time of triage (low blood pressure; hypoxia; mental status changes; clear signs of hypoperfusion) are generally classified as ESI 1 patients and prioritized for immediate treatment, diagnosis, and stabilization. Thus, they do not require further differentiation prior to initiation of treatment. Protocols should be created to streamline the care of these patients and dedicated resuscitation and Emergency Department-based Resuscitation Spaces linked to an ED-Intensive Care Unit appear to improve outcomes [22]. Less critically ill patients on triage presentation, triaged as ESI 2 or 3, such as those recruited for this study, present different problems centered on rapid detection and initiation of care when compared to the more critically ill whose main issues are optimization of care. By definition, patients triaged as ESI 2 and 3 are assumed to be stable enough to wait 20 minutes to 2 hours for care. However, patients with potential sepsis are dynamic, can be rapidly changing, and when severe enough, sepsis becomes a time-sensitive disease [23–27]. Patients with sepsis admitted to the hospital from the ED who are hemodynamically stable during their ED treatment have increasing mortality as initial lactate levels move from normal (< 2 mmol/L) to mid-range (2-3.9 mmol/L) to a level consistent with shock (≥ 4 mmol/L), with mortality increasing from 8.9 to 16 % to 32.9 %, respectively [28]. Based on these data as well as results of other studies, early measurement of serum lactate in patients with suspected sepsis has become standard of care in sepsis protocols around the world. However, additional data to optimize risk stratification of these patients are still needed and that need provided the rationale for trialing the NICOM device to measure non-invasive hemodynamic variables at triage and two hours later. This need is highlighted by research demonstrating that validated measures of ED crowding, which is a rapidly increasing problem in United States EDs, do not impact time to fluids or antibiotics in patients presenting with obvious acute organ dysfunction but do directly impact these process measures in less sick patients admitted from the ED to the hospital with sepsis [27]. Since the mortality of this cohort of patients approached 15 %, it is clear that other methods of early detection are needed. In the COMMIT trial, Shapiro and colleagues randomized 64 normotensive sepsis patients with a lactate level between 2 and 4 mmol/L to volume resuscitation guided by non-invasive cardiac output monitoring vs. standard clinical care [29]. Using this strategy, they found no differences in change in SOFA score but did find that the intervention group received more intravenous fluid during the intervention period. This study differs from our study, which enrolled patients on average within 10 minutes of ED triage, in that patients were required to be enrolled within 4 hours of ED presentation and 2.5 hours of meeting eligibility criteria.

### Limitations

This study should be viewed in the context of several limitations. The study population included a small convenience sample of 50 patients based on the availability of study personnel. Study personnel enrolled patients primarily during the evening hours on both weekdays and weekends. It is possible that patients who come to the ED during morning hours or overnight, meet 2 triage SIRS criteria, and have a chief complaint consistent with infection, have different risks for sepsis from the study population. It is also possible that the cohort of patients studied was not sufficiently sick from a sepsis severity perspective to show the benefit of early post-triage hemodynamic monitoring using the NICOM device since only 11/50 (22 %) patients were found to have acute organ dysfunction (as defined by the 2nd International Sepsis Definitions conference) [30]. Specifically, we may not have included sufficient patients with early myocardial depression of sepsis to demonstrate a role for the NICOM device in risk assessment. Additionally, the authors chose to omit measuring a change in NICOM cardiac SV as a surrogate for the patient’s fluid responsiveness during the passive leg raise (PLR) maneuver. A PLR is performed by changing the head of bed from a 45° angle to a flat position and moving the legs and feet from a flat position to 45 degrees of elevation [21]. It was believed that a practical use of the NICOM in the triage waiting area should include measurements that can be just as quickly obtained as a set of vital signs. The initial time from study enrollment to first NICOM reading routinely took less than ten minutes. If the NICOM was incorporated into the triage procedures in a busy ED, the PLR would be cumbersome to perform and potentially delay door-to-doctor time. However, the approach taken in the study protocol may have limited early detection of potential sepsis patients by not assessing fluid responsiveness as early as possible. Patients in the study remained in the post-triage waiting room during the two hours between NICOM values and, therefore, did not receive volume resuscitation during the study period. Our samples were evenly distributed except for one outlying value in all but the HR analysis (for CO, CI, SV) and given the sample sizes of our subgroups these outlying values may have impacted our analysis. We cannot exclude that changes in NICOM values between time zero and time 2 hours may reflect “noise” emanating from the measuring device. However, very clear trends in changes in NICOM values were seen: decreasing heart rate, increasing stroke volume, and slight decreases in cardiac output and cardiac index. Further, it is unlikely that these observations reflect a “white coat phenomenon.” Patients were seen prior to enrollment by a triage nurse, not a physician however the white coat phenomenon can occur with contact with any category of health care provider. More likely, the exertion of walking into the hospital and the anxiety about their medical condition contributed to the initial elevation in heart rate noted at triage and on time zero NICOM readings. Lying still and decreased stress will lead to decreased heart rate and this is reflected in the second NICOM readings. In addition, our patients did not receive fluid boluses between time zero and time two-hour NICOM measurements and this may have contributed to our lack of detection of NICOM as a tool for risk stratification. In fact, the NICOM device may be most helpful for fluid management as a study by Oord and colleagues demonstrated that a 500 ml fluid bolus did not reveal SV changes while a 1000 ml bolus was sufficient to demonstrate hemodynamic responses [31]. Finally, questions have been raised about the sensitivity and specificity of easily implemented, non-invasive methods to assess fluid responsiveness such as the NICOM device when compared to left ventricular outflow tract echocardiography, which is considered the non-invasive gold standard correlating most accurately with the values obtained from PAC [32].

### Future directions

Future work informed by this study should incorporate PLR performed immediately after the time zero NICOM values and the change in SV from time zero and two hours into the data. If patients have a positive response to PLR demonstrating fluid responsiveness, then they should have immediate treatment, including fluid bolus, initiated. Studies comparing NICOM CO measurements in a baseline semi-recumbent position versus changes observed when the PLR is performed and in hemodynamic measurements in patients undergoing the PLR versus those getting a fluid bolus have shown conflicting results [33–36]. Generalization of some of these results is limited by the study populations being limited almost exclusively to patients in intensive care units. Other vital signs and their trends including blood pressure, shock index (HR/SBP), and RR have been shown to be important in risk stratification of undifferentiated patients in the ED [37–39] and novel machine learning techniques integrating these values with lactate, other inflammatory and perfusion markers, along with hemodynamic measurements may yield better results in future studies.

## Conclusions

In conclusion, this study found that NICOM CO and CI, obtained at two separate time points after ED triage, do not help with early differentiation of patients at risk for sepsis who do not have obvious organ dysfunction at triage. This lack of differentiation occurred when assessed in relation to their disposition from the ED or the presence or absence of infection. While some significance was observed when comparing changes in CO, CI, SV, and HR to the presence of infection, this association was driven primarily by the significant differences in the mean HR of patients who have an infectious source. NICOM appears to have a role in the hemodynamic monitoring of sepsis patients during proximal resuscitation but the results of our study do not support its use as a risk stratification tool. Furthermore, even though the study found a significant difference in the mean HR of patients who were admitted versus those who were discharged, a serum lactate still served as a better predictor of patient admission from the ED.

## Appendix

Appendix: 50 Chief Complaints from the Advanced Triage Protocol

1. Abdominal Distention
2. Abscess
3. Chills
4. Cough
5. Chest Pain
6. Cerebral Vascular Accident/Transient Ischemic Attack
7. Dehydrated
8. Diarrhea
9. Diff Breathing
10. Dizzy
11. Dyspnea
12. Fatigue
13. Fever
14. Genitourinary
15. Headache
16. Hematuria
17. Hemoptysis
18. Hyperglycemia
19. Hypoglycemia
20. Infection
21. Infection, Extremity
22. Infection, Eye
23. Infection, Face
24. Infection, Foot
25. Infection, Hand
26. Infection, Joint
27. Infection, Leg
28. Infection, Sinus
29. Infection, Wound
30. Inflammation
31. Jaundice
32. Lethargy
33. Lightheadedness
34. Low Blood Pressure
35. Mental Status Change
36. Nausea/Vomiting
37. Nausea/Vomiting/Diarrhea
38. Neutropenia
39. Pain, Abdomen
40. Pain, Chest
41. Pain, Flank
42. Rash
43. Shortness of Breath
44. Sore Throat
45. Swell
46. Upper Respiratory Signs
47. Urinary Tract Infection
48. Vomiting
49. Weak
50. Wound Check
